# Does Antibody Stabilize the Ligand Binding in GP120 of HIV-1 Envelope Protein? Evidence from MD Simulation

**DOI:** 10.3390/molecules26010239

**Published:** 2021-01-05

**Authors:** Shalini Yadav, Vishnudatt Pandey, Rakesh Kumar Tiwari, Rajendra Prasad Ojha, Kshatresh Dutta Dubey

**Affiliations:** 1 Center of Informatics and Department of Chemistry, School of Natural Sciences, Shiv Nadar University, Uttar Pradesh 201314, India; sy776@snu.edu.in; 2 Department of Physics, Deen Dayal Upadhyay Gorakhpur University, Uttar Pradesh 273009, India; vishnudattpandey127@gmail.com (V.P.); drrkt@yahoo.in (R.K.T.); rp_ojha@yahoo.com (R.P.O.)

**Keywords:** HIV-entry inhibitor, MD simulations, free energy calculations, conformational mechanism

## Abstract

CD4-mimetic HIV-1 entry inhibitors are small sized molecules which imitate similar conformational flexibility, in gp120, to the CD4 receptor. However, the mechanism of the conformational flexibility instigated by these small sized inhibitors is little known. Likewise, the effect of the antibody on the function of these inhibitors is also less studied. In this study, we present a thorough inspection of the mechanism of the conformational flexibility induced by a CD4-mimetic inhibitor, NBD-557, using Molecular Dynamics Simulations and free energy calculations. Our result shows the functional importance of Asn425 in substrate induced conformational dynamics in gp120. The MD simulations of Asn425Gly mutant provide a less dynamic gp120 in the presence of NBD-557 without incapacitating the binding enthalpy of NBD-557. The MD simulations of complexes with the antibody clearly show the enhanced affinity of NBD-557 due to the presence of the antibody, which is in good agreement with experimental Isothermal Titration Calorimetry results (*Biochemistry* **2006**, *45*, 10973–10980).

## 1. Introduction

**Scheme 1:**
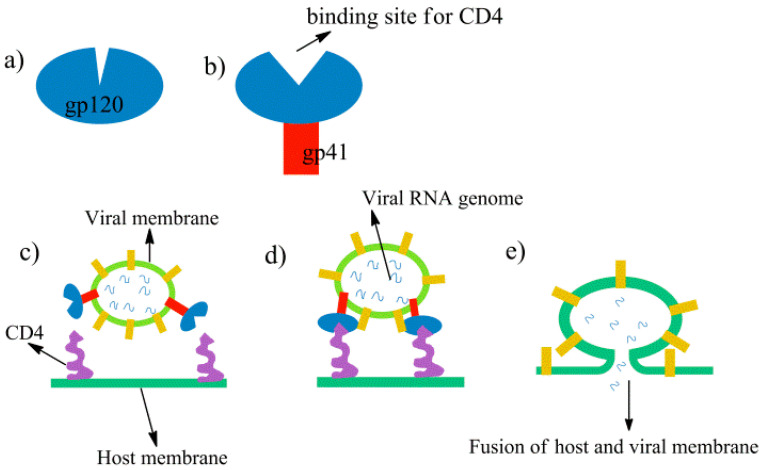
A schematic representation of HIV-1 entry and fusion of virion into the host membrane. (**a**) a schematic representation gp120, (**b**) a schematic representation of gp41 binding with CD4 and (**c**–**e**) attachment and fusion of virus with host membrane

The pathogenic importance of human immunodeficiency virus type-1 (HIV-1) is well known [1,2,3]. The HIV-1 infection is caused by the fusion of viral and host membranes in many sequential steps which are initiated by the non-covalent attachment of two regulatory subunits of HIV-1 envelope protein, gp120 and gp41 [4]. These sequential steps of viral–hosts fusion can be comprehended by Scheme 1.

**Figure 1:**
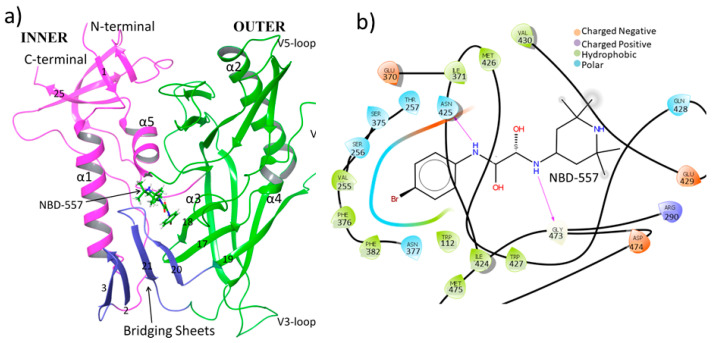
(**a**) Three-dimensional structure of gp120 with structural topology. The inner domain is shown in pink, the outer domain is shown in green, while bridging sheets are shown in blue colors. Numbering of β-sheets is taken from ref. [11] and only a few of them are shown to maintain minimalism in the figure. (**b**) Two-dimensional ligand interaction diagram of gp120 from the crystal structure [20]; H-bonds are shown by pink arrows.

Initially, each unit of gp120 protein is arranged into homotrimeric fashion and forms a closed cavity mushroom head-type system, as shown in Scheme 1a, while the non-covalent binding of the gp41 protein with gp120 triggers some conformational changes that open a binding cavity for CD4 receptors and forms a complete mushroom shape, as shown in Scheme 1b [5,6]. The HIV-1 virion contains the RNA genome encapsulated by viral membrane where gp120.gp41 complexes are attached to the membrane surface and are exposed to the CD4 protein receptors of host membrane, as shown in Scheme 1c [7,8]. The docking of the virion via gp120 to the host membrane gives rise to the conformational rearrangement in the envelope protein that pulls the viral membrane close to the CD4 subunits of host membrane [9], as shown in Scheme 1d. The binding of CD4 receptors via gp120 protein leads to more conformational changes and finally the fusion of host and viral membrane takes place, as shown in Scheme 1e. As is clear from the Scheme 1, the gp120 is central to viral entry via binding to gp41 and CD4; it turns out to be an important topic in the targeting of HIV-1 infections. The structural topography of gp120 revealed by X-ray crystallography shows that two domains comprising a mixture of α- and β- motifs constitute the structure of gp120: the inner domain (residues 1–117, 206–257, 475–492) which interacts with gp41 protein, and the outer and less conserved domain (residue 437–473, 263–417) which interacts with CD4 and co-receptor (CR) proteins (see Figure 1 for the structure of gp120) [10,11]. These two domains are connected via four bridging sheets (residues 418–430). Since the binding of gp120 with CD4 receptor proteins is the crucial step during HIV-1 infections, therefore, the site of CD4 binding in gp120 (Scheme 1b) becomes a productive target for HIV-1 inhibition [12,13,14,15]. The details and the recent development of these HIV-1 entry inhibitors targeting the gp120 proteins can be found in some informative review articles where we observe that the majority of the HIV-1 entry inhibitors are either BMS-378806 and its analogous inhibitors or NBD-556 and its derivatives [16,17,18]. Interestingly, these two inhibitors target same receptor but their mechanism is entirely different. An experimental study of Schön et al. shows that the binding mode and antiviral properties of these inhibitors are governed by their binding thermodynamics [19]. In the same study, the authors show that BMS-378806 has very weak binding enthalpy and does not compete with the CD4-coreceptor; on the other hand, NBD is a CD4-coreceptor competitive inhibitor and reflects similar conformational flexibility and binding thermodynamics as the CD4-coreceptor. However, the means by which the binding of small molecules such as NBD instigates the conformational flexibility in gp120 remains an enigma. The current study provides a theoretical explanation of the mechanism of the conformational dynamics instigated by the inhibitor.

The instability of gp120 protein, when separated from its membrane anchored part, is a fundamental problem in obtaining the crystal structure of gp120 [4]. Therefore, most of the structures of gp120 were obtained with core domain bound antibody, even in the presence of HIV-1 entry inhibitors. This often raises a possibility that the binding of HIV-1 inhibitor in the presence of antibody may influence the interactions of the inhibitor. Does antibody presence affect the mechanism of the HIV-1 inhibitor? If, yes then how? This study provides a valuable insight on these questions using Molecular Dynamics (MD) Simulations and free energy calculations. The study of Schön et al. provides a small hint that the presence of antibody may enhance the ligand binding and, thus, the efficacy of the inhibitor; however, a comprehensive investigation is still missing. We have provided a mechanistic insight into and a comprehensive study of the effect of antibody on the binding of HIV-1 entry inhibitors using sufficiently long molecular dynamics (MD) simulations with a typical example of NBD-557 with gp120. In order to successfully conduct such a study, we firstly emphasized the NBD-557 interactions and its binding thermodynamics without antibody and then compared the NBD-557 interaction and its binding in the presence of antibody.

## 2. Methods

### 2.1. System Setup

The initial structure was taken from the protein data bank (PDB id: 4DVR) [20], which contains gp120 core, ligand NBD-557, and antibody Fab 48d. The original structure, 4DVR, has several missing loops which were modelled by MODELLER. Since we have focused on the role of antibody on NBD-557 binding, we prepared two systems; one by extracting antibody and constructing a complex of ligand NBD-557 with gp120 only, and the second as ligand NBD-557 with gp120 and antibody. The protonation state of Histidine residues was calculated by propka 3.0 [21]. The pKa value for His216 was 6.72; therefore, we kept it in a doubly protonated state, i.e., HIP, and the rest of the Histidines were kept in either HIE (epsilon protonation) or HID (delta protonation) state depending upon the H-bond possibility with nearby residues. The missing hydrogens were prepared with the Leap module of Amber 14 with Amber ff14SB forcefield parameters [22]. Since the parameters for NBD-557 were not available in the Amber library, we used the antechamber module of Amber to generate the parameters for NBD-557. The partial atomic charges and missing parameters for the NBD-557 were obtained from the RESP [23,24] (restrained electrostatic potential) method using HF/6-31G* geometry optimization in the Gaussian 09 package. We used five and two Cl^−^ ions to neutralize the NBD-557 with gp120 only complex and the NBD-557 with gp120 and antibody complex, respectively. Finally, the resulting systems were solvated in a rectangular box of TIP3P [25] waters extending up to minimum cutoff of 10 Å from the protein boundary.

### 2.2. MD Simulations

After proper parameterizations and setup, the resulting system’s geometries were minimized (5000 steps for steepest descent and 10,000 steps for conjugate gradient) to remove the poor contacts and relax the system. The systems were then gently annealed from 10 to 300 K under canonical ensemble for 50 ps with a weak restraint of 5 kcal/mol/Å^2^. Subsequently, the systems were maintained for 1 ns of density equilibration under isothermal–isobaric ensemble at the target temperature of 300K and the target pressure of 1.0 atm using a Langevin-thermostat [26] and a Berendsen barostat [27] with a collision frequency of 2 ps and a pressure relaxation time of 1 ps, and with a weak restraint of 1 kcal/mol/Å^2^. Thereafter, we removed all restraints applied during heating and density dynamics and further equilibrated the systems for ~3 ns to obtain well settled pressure and temperature for conformational and thermodynamical analyses. This was followed by a productive MD run of 100 ns for each system. We used three different replicas starting from different initial velocities each for 100 ns. Therefore, we performed a total of 300 ns simulations including all three replicas. During all MD simulations, the covalent bonds containing hydrogen were constrained using SHAKE [28], and a particle mesh Ewald (PME) [29] was used to treat long-range electrostatic interactions. We used an integration step of 2 fs during the entire simulations. All MD simulations were performed with the GPU version of the Amber 14 package [30].

### 2.3. Clustering of the Trajectories

The energetics of ligand binding using end point methods such as MMGB/SA (Molecular Mechanical Generalized Boltzmann/Surface Area) depend upon the selection of MD frames [31]. Therefore, we have extracted the most populated trajectories from the MD simulations of all three replicas using clustering of trajectories, which provides an accurate means to represent the most statistically significant structures during MD sampling. Firstly, we seamed all replicas together using the cpptraj module of Amber, which generated 30,000 frames (300 ns) for each complex. Thereafter, we performed clustering of MD trajectories using the hieragglo algorithm implemented in Cpptraj. This produced 10 different clusters of trajectories (see page S6 of Supplementary Materials) where the most populated trajectories were nearly 100 ns long, and all analyses were performed for these most populated trajectories. The results of the clustering are shown in the Supplementary Materials.

### 2.4. Free Energy Calculations

As stated by Schön et al., the antiviral properties of HIV-1 entry inhibitors are reflected by the thermodynamics of these inhibitors; we used the MM-GB/SA method [32,33] for the calculations of thermodynamic parameters and free energy of binding. The principles of these methods are well established [34,35] and have been successfully applied for the class II fusion viruses in the previous studies [36,37,38]. For all MMGBSA calculations, we used the most populated MD trajectories obtained from clustering of trajectories (100 ns). Note that all energetics are calculated for the most populated frames of MD trajectories obtained from cluster analysis, as described above. All water molecules and the chloride ions were stripped from the trajectory prior to the MMPBSA analysis. The dielectric constant for the solute and the surrounding solvents were kept at 1 and 80, respectively. The MMPBSA calculations were performed on the most populated trajectories obtained from cluster analysis. All analysis of trajectories was done with the Cpptraj module of Amber14. VMD 1.6.7 [39], Chimera-1.5 [40] graphical programs were used for the visualization. The hydrogen bonds and its occupancies were calculated by VMD for production trajectories, where we kept the donor–acceptor distance as 3.0 Å and the angle cut-off as 20°.

## 3. Results and Discussion

We begin with the conformational properties of gp120 complex with ligand NBD-557 when the antibody is not binding; thereafter, we discuss the conformational properties of the gp120 complex with ligand NBD-557 when the antibody is binding.

### 3.1. MD Simulation of gp120 with NBD-557

The RMS deviations (root mean square) with simulation time for the MD simulation of gp120 with NBD-557 are constant, which shows a converged MD trajectory (see Figure S1 in Supplementary Materials for the most populated 100ns, and Figure S5 for the overall trajectory). We also simulated gp120 only in the absence of NBD-557 to study the effect of ligand binding. The RMS deviations of gp120 in the presence of ligand (NBD-557) are slightly more converged vis-a-vis gp120 in the absence of ligand, which shows that ligand binding stabilizes the simulation.

**Figure 2:**
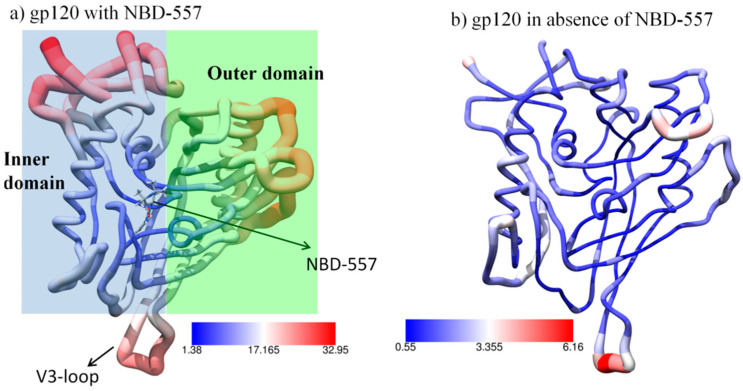
The root mean square fluctuations for gp120 shown in a worm model. The thickness of the worm radius represents the value of fluctuations (**a**) with NBD-557; the inner and outer domains are highlighted by blue and green transparent rectangles (**b**) without NBD-557. The values of RMSF (Å^2^) are shown by colour keys in each figure.

**Figure 3:**
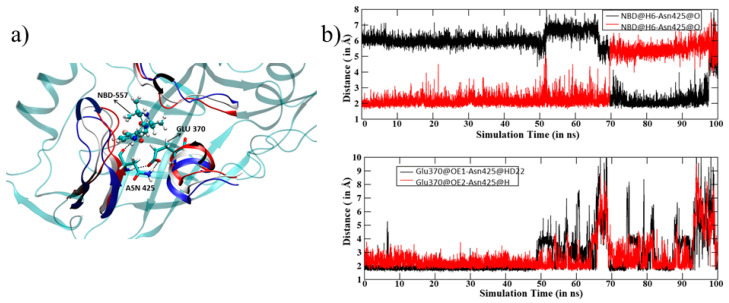
(**a**) Key residues interacting with NBD-557. Notice the movement of structural motifs surrounding ligand indicated by arrows (blue represents initial, white shows mid and red shows the orientations at the last step of MD simulations. (**b**) The distance vs. simulation time for 100 ns of MD simulation for key H-bonds (top) and the distance between Glu370 and Asn425 (bottom). Note a constant and strong interaction of NBD-557 with Asn425. Note that all results are reported for the most populated trajectories of 100 ns.

The root mean square fluctuations (RMSF) of gp120 with and without NBD-557, as shown in Figure 2, very clearly emphasize the effect of ligand binding. We can see that the conformational flexibility of gp120 is very high in the presence of ligand vis-a-vis when ligand is not binding. More importantly, the functional elements (the inner domain that interacts with gp41, the outer domain that interacts with CD4 and the CR proteins of the target cells) show the highest flexibility. The increased conformational flexibility at gp41 and CD4+CR binding interfaces due to inhibitor binding substantiates the fact that NBD-557 is a CD4-mimetic inhibitor, as similar flexibility in gp120 is supposed to be induced by binding of the CD4 co-receptor [5]. The increased flexibility due to NBD-557 may raise some obvious questions as to how the binding of a small molecule can trigger the conformational dynamics in gp120, as well as the driving force for this phenomenon. Therefore, we thoroughly monitored the interactions of NBD-557 with active site residues and found that it is interaction between NBD-557 and Asn425 that drives the flexibility of gp120 with course of simulations (see Figure 3).

The ligand NBD-557 contains two amine hydrogens situated at two different positions (see Figure 1b) which are prone to forming hydrogen bonds with backbone oxygen of Asn425. This Asn425 forms a strong hydrogen bond with another residue, Glu370. Interestingly, these two residues are part of the bridging sheets (236–250) and outer domains; therefore, NBD-557 acts as a bridge between different functional subunits of gp120. During the course of MD simulations, the amine hydrogens of NBD-557 exchange their positions to form a strong bond with backbone oxygen of Asn425 (note the exchange of H-bonding from 70–100 ns of MD simulations and a strong correlation between the upper and lower plots during 50–100 ns of simulations; we can easily see that the fluctuation in the lower graph is strongly correlated with H-bond exchange in the upper plot), which in turn produces local dynamics in Asn425 and thus in Glu370. Interestingly, Asn425 and Glu370 are residues situated at bridging interfaces between the inner and outer domains and a disturbance at this site produces fluctuations at the inner and outer domain end, as seen in Figure 2a. For further validation of this mechanism, we performed another MD simulation of gp120 in the absence of ligand and found a drastic decrement in thermal fluctuations in gp120 (see Figure 2b). In this case, the binding between the Asn425 and Glu370 is rather weak due to absence of ligand, which might be reason for the conformational elasticity of gp120 (see Figure S2 for Asn425-Glu370 distance). The lack of thermal fluctuations in the absence of NBD-557 strongly supports the ligand instigated dynamics in gp120, which are factually caused by the interaction between NBD-557 and Asn425.

### 3.2. Thermo-Chemistry of gp120 Complex with NBD-557

Schön et al. [19] reported the thermodynamics of binding of NBD-556 and NBD-557 with gp120 using ITC (Isothermal Titration Calorimetry) [19]. They found the change in enthalpy during binding of NBD-556 (same as NBD-557) to be −24.50 kcal/mol, and this can be used to benchmark our simulated structure. Therefore, we calculated the change in enthalpy using the MMGBSA method for NBD-557 binding, as shown in Table 1. The computed change of enthalpy is −25.16 ± 3.24 kcal/mol and is in good agreement with the experimental results [19]. The theoretical calculation shows that the van der Waals interactions (−36.94 kcal/mol) play a dominating role in enthalpic change during binding. The electrostatic contribution is rather weak and is quite expected as NBD-557 forms just two hydrogen bonds with Glu370 and Asn425. Furthermore, the interaction energy due to solvation presents an unfavourable contribution.

**Figure 4:**
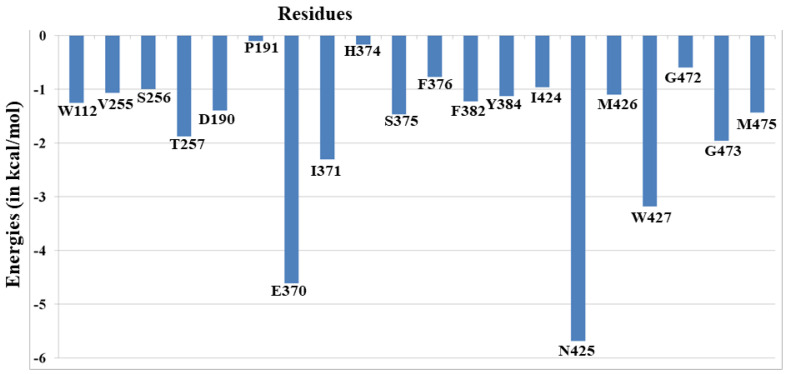
The residue interaction map for NBD with gp120 for the most populated trajectories of 100 ns.

To further emphasize the gp120–ligand interactions, we calculated the residue interaction map, as shown in Figure 4. The residue interaction map calculated by MMGBSA is very informative as non-polar and hydrophobic interactions cannot be analyzed in a similar manner to H-bond and salt-bridges. We can see that Glu370 and Asn425 interact strongly with NBD-557 due to the H bond, as shown in Figure 4. The ligand is mostly in contact with several hydrophobic residues such as Trp112, Val255, Ile371, Phe376, Phe382, Ile424, Met426, Gly472, Gly473 and Met475. These hydrophobic and non-polar residues contribute significantly to the total favourable change of enthalpy during binding. Interestingly, similar interactions were predicted in the crystal structure of NBD-557 with gp120 [20]; therefore, the calculated residue interactions map provides a theoretical validation and shows the stability of such interactions during 100ns of MD simulations.

### 3.3. Dynamics of the In-Silico Mutant Asn425Gly

**Figure 5:**
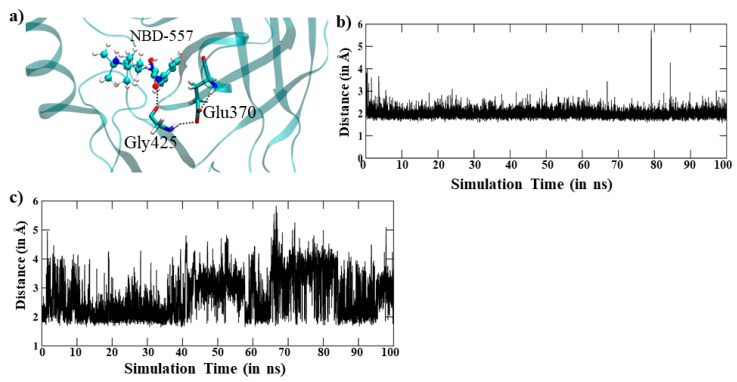
Results from the MD of N239G mutant. (**a**) NBD-557 binding in the gp120 binding pocket. (**b**) The distance *vs*. simulation time for the H-bond between amine hydrogen of NBD-557 and backbone oxygen of Gly425. (**c**) Distance vs. time for the H-bond between Gly425 and Glu370. Note that all results are reported for the most populated trajectories of 100 ns.

Since NBD-557 interacts strongly with Asn425, we performed in-silico mutation of Asn425 to Gly425 to monitor the effect of in-silico mutations. Interestingly, the ligand remains quite stable. In the Asn425Gly mutant, the one of amine hydrogen (H1--N) of NBD-557 forms a very strong hydrogen bond with backbone oxygen of Glycine and does not show the switching of position, as seen in WT complex (see Figure 5a,b). However, the Gly425 interaction with Glu370 is not as strong as in WT complex. The change in total energy (enthalpy) of binding is more favourable vis-a-vis WT complex, which clearly indicates that NBD-557 is a strong binder for the mutant complex (see Table S1).

### 3.4. Effect of Antibody Binding on gp120 Interaction with NBD-557

**Figure 6:**
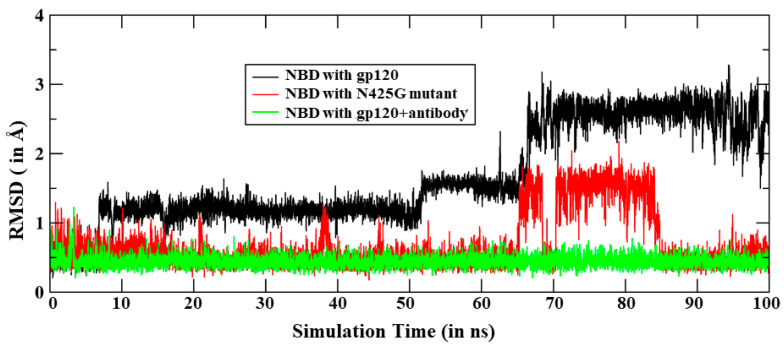
The RMS deviations for NBD-557 in all three MD simulations, i.e., with gp120, in Asn425Gly mutant and with antibody. Note that all results are reported for the most populated trajectories of 100 ns.

**Figure 7:**
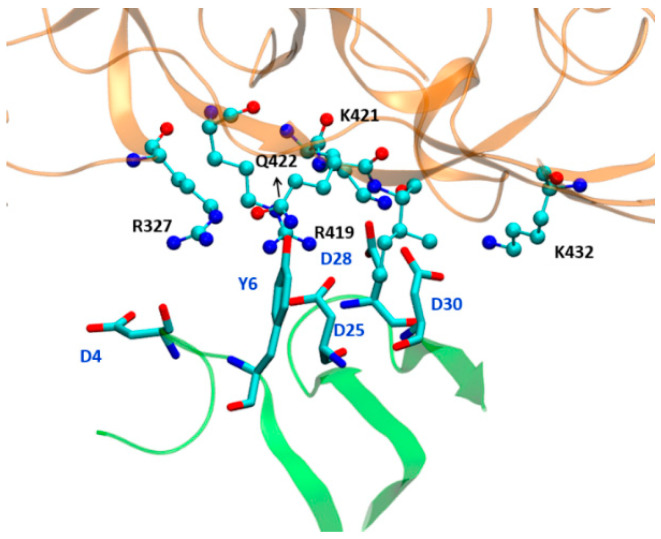
The orientation of interfacial residues of antibody and gp120 protein. The part of gp120 is shown in golden while antibody is shown in light green colour.

The study by Schön et al. [19] indicates that the binding of antibody increases the binding of NBD-556 and NBD-557. They found that the enthalpy change on the binding of ligand increases from −24.5 kcal/mol to −28.9 kcal/mol in the presence of antibody. Therefore, we performed a 200 ns long MD simulation of the gp120 complex with NBD in the presence of antibody and we have calculated the change in enthalpy on NBD-557 binding. Our theoretical calculations strongly support the finding of Schön et al. that antibody presence enhances the binding affinity of NBD-557. A comparison of RMS deviations for NBD-557 in WT complex, R425G mutant and gp120 complex with NBD is shown in Figure 6. We can easily notice the stability of the NBD-557 in gp120 with antibody relative to WT and mutant. The change in enthalpy of binding is shown in Table 2, which quantitatively supports the enhanced binding affinity of drug due to antibody bonding. To decipher the rationale for the increased affinity due to antibody, we thoroughly studied the interactions between gp120 and antibody. We notice that residues 6–61 of antibody are proximal to the gp120 interface and may interact with gp120. The interface between antibody and gp120 are rich of acidic residues such as Asp6, Asp28, Asp25, Asp30 of antibody, and Arg327, Arg419, Lys421 and Lys432 at the gp120 site, which provide stability to the gp120 and antibody complex. The interaction energy of these residues, calculated by residue decomposition of MMPBSA, is shown in Table 3. These data quantitatively confirm that there are very strong acid–base pair interactions between gp120 and antibody interfaces such as Asp25-Arg419, Asp28-Arg419, Asp28-Lys421, Asp30-Lys421 and Asp30-Lys432 (See Figure 7 and Table 3).

**Figure 8:**
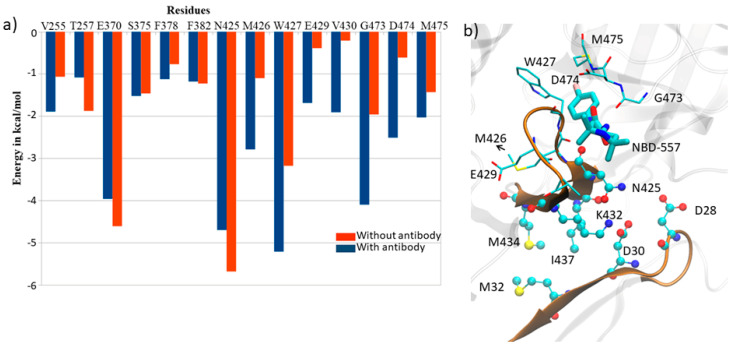
(**a**) A comparative residue interaction map between gp120 and inhibitor NBD-557 in the presence and absence of antibody. (**b**) The representation of residues at the bridging sheet and antibody.

The results shown above provide compelling evidence that the presence of antibody in gp120 stabilizes the complex via acid–base pair interactions at the binding interface; however, whether the antibody affects the drug binding is still unknown. Therefore, we calculated the change in enthalpy upon ligand binding in the presence and absence of antibody (See Table 1 for NBD-557 binding enthalpy in the absence of antibody). Our calculations, shown in Table 4, show that the presence of antibody not only stabilizes the gp120 complex via acid–base pair interactions, but also increases the binding of ligand. We can see that, in the absence of antibody, the change in enthalpy due to ligand binding is −25.16 kcal/mol, while, in the presence of antibody, it significantly increases to −33.57 kcal/mol. To investigate the rationale for the increased affinity, we thoroughly monitored the MD trajectories and the residues of gp120 participating in ligand binding and antibody-gp120 binding. Figure 8a shows a comparative interaction map due to antibody binding. It is quite clear that the binding of antibody increases the interactions with NBD for some residues, e.g., Met436, Trp427, Val430, Gly473, Asp474, which provide stability to the binding of NBD-557. As Figure 8b shows, these residues reside on the bridging sheet 20 (see Figure 1b and Ref. [11] for structural topology), which is an interfacial loop interacting with the antibody; therefore, these residues show conformational rearrangement, which might cause increased interactions with NBD on antibody binding.

## 4. Conclusions

NBD-557 is a CD4 mimetic and its competitive receptor is believed to induce similar conformational changes to the CD4 receptor. Our MD simulations clearly show that NBD-557 induces large conformational flexibility, particularly in the outer and inner domains. The absence of such flexibility in the apo state of gp120 further substantiates the fact that such dynamics are mainly instigated by the inhibitor, which is in good agreement with experimental observations. A thorough inspection of the simulated trajectories shows that the interaction of NBD-557 with Asn425 is a key factor that choreographs the substrate ligand-induced conformational flexibility in gp120. The role of Asn425 was further supported by the MD simulations of N425G mutant. We have found that the N425G mutation increases the binding affinity but it does not induce the flexibility in gp120 due to a lack of flexible side chain interaction with NBD-557. The thermodynamical parameters of NBD-557 in the presence of antibody show enhanced binding enthalpy of inhibitors. We found that antibody possesses many acidic residues at its gp120 facing interface, which interacts strongly with basic residues of gp120, and thus provides additional stability to the gp120–NBD-557 complex, which, in turn, increases the binding affinity of the complex. Our results from the MD simulations of the gp120 with antibody (Fab80) provide a theoretical validation that the antibody stabilizes the gp120 via crucial acid–base pairs at the binding interface between antibody and gp120. The binding free energy calculations quantitatively show that antibody binding increases the change in total binding energy via some conformational rearrangement in bridging sheet β20, which forms the active site for NBD-557 binding.

In summary, the present study provides a comprehensive molecular mechanism of NBD-557 binding and the means by which it mimics conformational rearrangement by the CD4 co-receptor. Our study provides a theoretical validation of the fact that antibody stabilizes the gp120 and ligand binding. The mechanism of conformational changes instigated by NBD-557 and antibody can be useful for future studies for targeting HIV-1 entry.

## Figures and Tables

**Table 1:** Energy contribution when GP120 binds with NBD in the absence of antibody. Here, ΔE_VDW_ = change in van der Waals interaction on ligand associations, ΔE_EEL_ = change in electrostatic interaction on ligand associations, ΔE_polar_ = change in polar interaction on ligand associations, ΔE_nonpolar_ = change in nonpolar interaction on ligand associations. ΔG_gas_ = ΔE_VDW_ + ΔE_EEL_, ΔG_solv_ = ΔE_polar_ + ΔE_nonpolar_, ΔG_binding_ = ΔG_gas_ + ΔG_solv_.

Energy Contribution	Values (in kcal Mol)	Standard Error
∆E_VDW_	−36.94	2.19
∆E_EEL_	−5.22	1.93
∆E_NPOLAR_	21.33	1.71
∆E_DISPER_	−4.32	0.17
∆G_gas_	−42.16	3.04
∆G _solv_	17	1.67
**∆H_TOTAL_**	**−25.16**	3.24
**∆H_EXP_^ref^**	**−24.50**	

**Table 2:** Energy contribution when GP120 binds with NBD in Asn425Gly Mutant. All abbreviations have the same meaning as defined in Table 1.

Energy Contribution	Values (In kcal Mol)	Standard Error
∆E_VDW_	−46.19	3.23
∆E_EEL_	−14.88	3.3
∆E_NPOLAR_	29.65	2.52
∆E_DISPER_	−5.28	0.32
∆G_gas_	−61.07	5.25
∆G_solv_	24.36	2.32
**∆H_TOTAL_**	−36.71	3.66

**Table 3:** The interaction between antibody and gp120 residues. Here antibody residue numbering starts from 1 and it is different from gp120 numbering.

Residues (Antibody)	Residues (gp120)	Energy (kcal/mol)
TYR6	ARG327	−3.928
TYR6	GLN422	−2.059
ASP25	ARG419	−18.346
ASP25	LYS421	−2.437
ASP28	PRO369	−1.679
ASP28	ARG419	−7.616
ASP28	LYS421	−10.152
ASP30	LYS421	−11.001
ASP30	ILE 423	−2.028
ASP30	LYS432	−7.517
MET32	MET434	−1.656

**Table 4:** The thermodynamical parameters for NBD-557 when binding with gp120 in the presence of antibody. All abbreviations have the same meaning as defined in Table 1**.**

Energy Contribution	Values (in kcal Mol)	Standard Error
∆E_VDW_	−43.94	2.16
∆E_EEL_	−14.12	2.19
∆E_NPOLAR_	29.40	1.46
∆E_DISPER_	−4.90	0.15
∆G_gas_	−58.07	2.75
∆G _solv_	24.49	1.40
**∆H_TOTAL_**	**−33.57**	2.38

## Supplementary Materials

The following are available online, The SI contains the details of the clustering, details of the residue wise interactions and RMS deviation plots.

## Sample Availability

Samples of the compounds are not available from the authors.
