# Supplementary material for: Does Antibody Stabilize the Ligand Binding in GP120 of HIV-1 Envelope Protein? Evidence from MD Simulation
Source: Molecules. 2021 Jan 5;26(1):239. doi: 10.3390/molecules26010239 (PMC7796314; doi:10.3390/molecules26010239)
Supplement: Supplementary file 1 [file molecules-26-00239-s001.pdf]

Supplementary Information

Does Antibody Stabilize the Ligand Binding in GP120 of HIV-1 Envelope Protein? Evidence from MD Simulation

Shalini Yadav[1#] ,Vishnudatt Pandey[2#],Rakesh Kumar Tiwari[2], Rajendra Prasad Ojha[2] and Kshatresh Dutta Dubey[1*],

[1]Department of Chemistry and Center of Informatics, School of Natural Sciences, Shiv Nadar University, Uttar Pradesh 201314, India.

[2]Department of Physics, Deen Dayal Upadhyay Gorakhpur University, Uttar Pradesh, India.

* Corresponding author, email: kshatresh@gmail.com, kshatresh.dubey@snu.edu.in

Table of Content:

Figure S1. The root mean square deviations of protein backbone, in presence and absence of the inhibitor, NBD-557………………………………… S2

Figure S2. The distance between the Glu370 and Asn425 when there is no substrate. We note that H-bond interaction is very week when there is no substrate…… S2

Figure S3. The H bond evolution for Glu370 and Asn425 for most populated 100ns……….. S3

Figure S4. The residue mapping of simulated structures and crystal structure …… S4

Figure S5. The RMSD for overall 300ns simulation for gp120 with ligand and antibody. ……..S4

Table S1. The percentage evolution of H-bonds between Asn425 and Glu370

Table S2. The thermodynamic parameters for the N425G Mutant……. S5

Table S3. Details of residuewise energy decompositions for interaction of NBD-557…. S5

Table S4. Details of residuewise energy decompositions for interaction of antibody and gp120…S5

Table S5-S7. Details of clustering for gp120 with NBD-557, N425 mutant, and gp120 with NBD in presence of antibody… S6

Figure S1. The root mean square deviations of protein backbone, in presence and absence of the inhibitor, NBD-557.

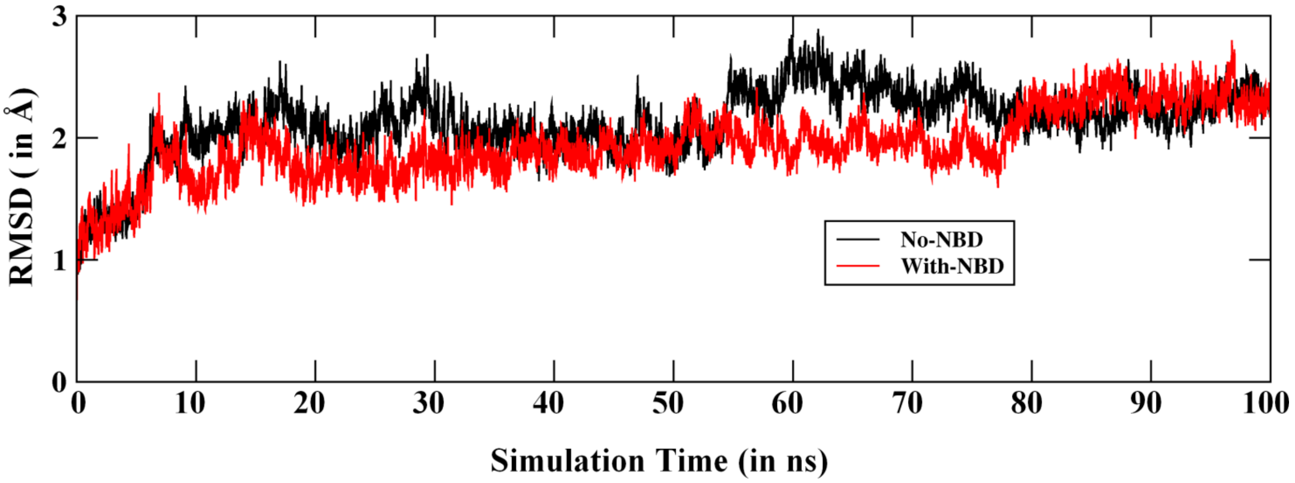

Figure S2. The distance between the Glu192 and Asn239 when there is no substrate. We note that H-bond interaction is very week when there is no substrate.

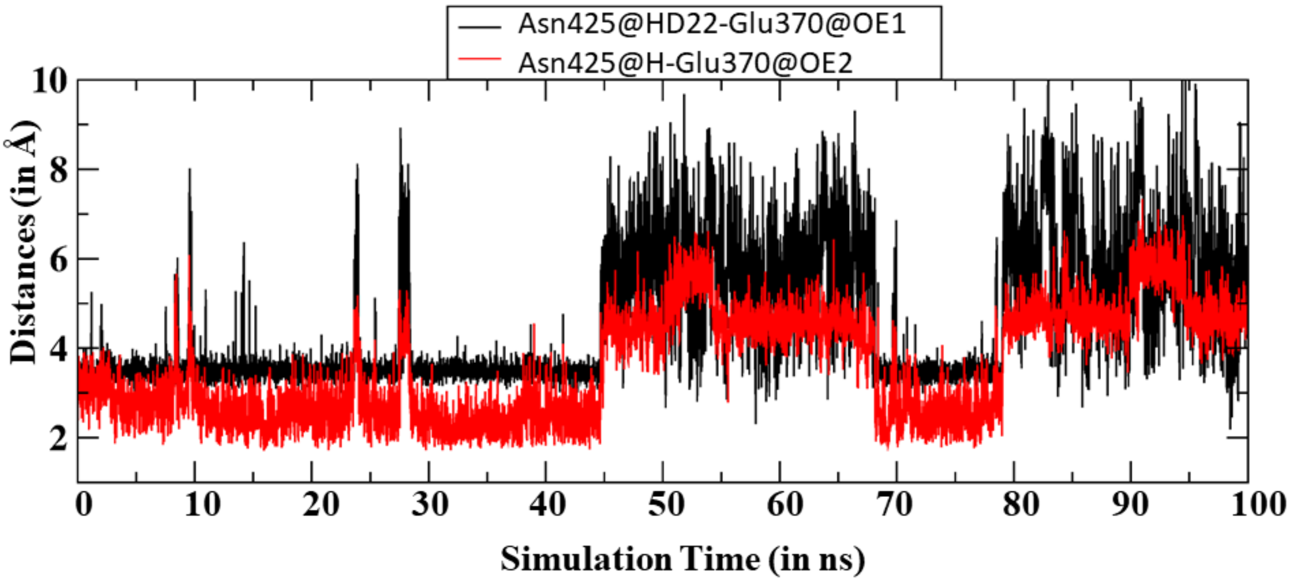

Table S1. The evolution of H-Bond for Glu370 and Asn425. H-bonds were calculated using VMD for most populated trajectory when NBD-557 binds gp120.

Found 2 hbonds.

**donor**	**acceptor**	**occupancy**
ASN425-Side	GLU370-Side	62.13%
ASN425-Main	GLU370-Side	3.56%

Figure S3. Hydrogen bond evolution with time for Glu370 and Asn425

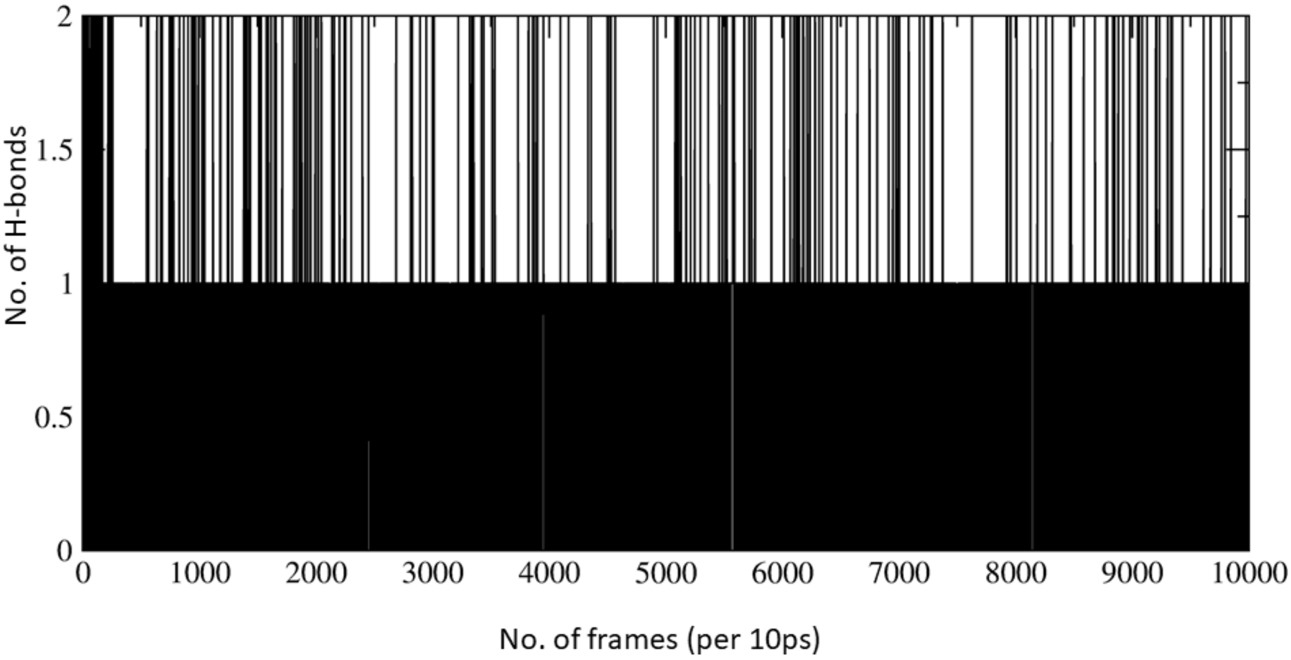

Table S2. The thermodynamic parameters for the N239G Mutant.

**Energy Contribution**	**Values (in kcal Mol)**	**Standard Error**
ΔE_{VDW}	-46.19	3.23
ΔE_{EEL}	-14.88	3.30
ΔE_{NPOLAR}	29.65	2.52
ΔE_{DISPER}	-5.28	0.32
ΔG_{gas}	-61.07	5.25
ΔG_{solv}	24.36	2.32
**ΔH_{TOTAL}**	**-36.71**	3.66

Figure S4. The residue mapping of simulated structures and crystal structure. The residues numbers in Table S3-S4 corresponds to simulated numbering.

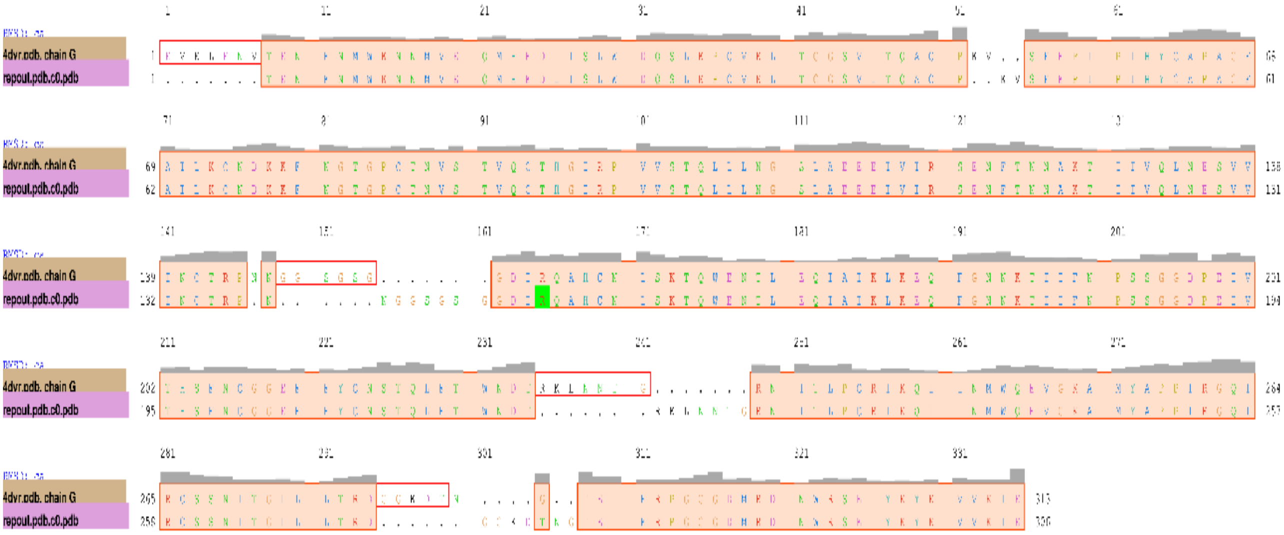

Figure S5. RMSD for entire 300 ns for gp120 with NBD-557 and with antibody.

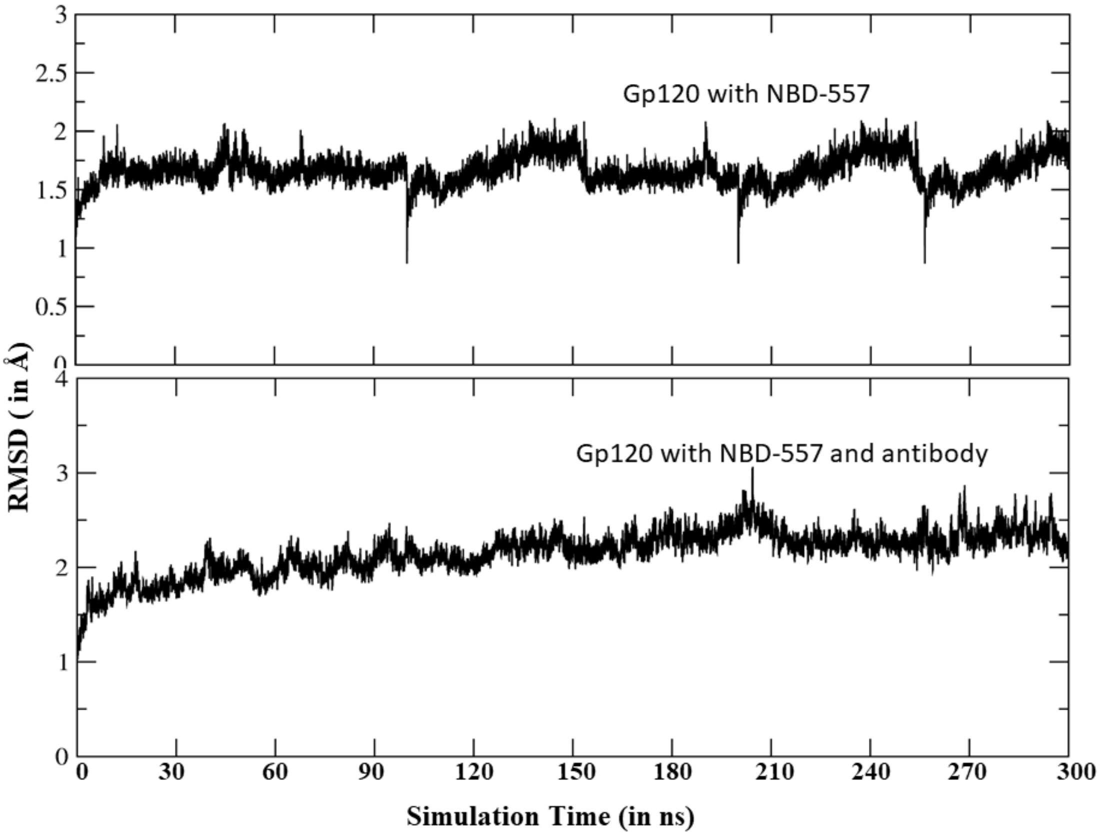

Table S3. Details of residuewise energy decompositions for interaction of NBD-557. All energy values are in kcal/mol. Data are shown as value ±standard error.

Total Energy Decomposition:

Resid 1	Resid 2	van der Waals	Electrostatic	Polar Solvation	Non-Polar Solv.	TOTAL
NBD 557	TRP 23	-0.800 ±0.238	-0.130 ±0.055	0.169 ±0.054	-0.492 ±0.140	-1.253 ±0.382
NBD 557	VAL 93	-0.658 ±0.199	0.198 ±0.217	-0.318 ±0.103	-0.291 ±0.092	-1.069 ±0.268
NBD 557	SER 94	-0.565 ±0.136	-0.360 ±0.100	0.134 ±0.063	-0.207 ±0.063	-0.998 ±0.240
NBD 557	THR 95	-1.146 ±0.240	-0.164 ±0.188	0.249 ±0.145	-0.817 ±0.145	-1.879 ±0.379
NBD 557	ASP 190	-0.636 ±0.173	-0.501 ±0.328	0.242 ±0.292	-0.499 ±0.152	-1.394 ±0.357
NBD 557	PRO 191	-0.139 ±0.021	0.150 ±0.039	-0.115 ±0.049	-0.000 ±0.001	-0.105 ±0.034
NBD 557	GLU 192	-2.357 ±0.322	-1.218 ±0.550	0.432 ±0.484	-1.472 ±0.160	-4.614 ±0.610
NBD 557	ILE 193	-1.195 ±0.254	0.073 ±0.062	-0.054 ±0.048	-1.127 ±0.199	-2.303 ±0.398
NBD 557	HID 196	-0.072 ±0.013	-0.235 ±0.055	0.138 ±0.051	0.000 ±0.000	-0.168 ±0.034
NBD 557	SER 197	-0.937 ±0.209	0.054 ±0.216	-0.157 ±0.145	-0.426 ±0.087	-1.465 ±0.338
NBD 557	PHE 198	-0.518 ±0.131	-0.229 ±0.102	0.099 ±0.040	-0.123 ±0.039	-0.771 ±0.189
NBD 557	PHE 204	-0.752 ±0.228	-0.283 ±0.079	0.286 ±0.043	-0.480 ±0.076	-1.229 ±0.257
NBD 557	TYR 206	-0.587 ±0.142	-0.242 ±0.200	0.036 ±0.162	-0.335 ±0.081	-1.128 ±0.245
NBD 557	ILE 238	-0.781 ±0.153	0.360 ±0.103	-0.036 ±0.049	-0.507 ±0.108	-0.964 ±0.202
NBD 557	ASN 239	-2.232 ±0.399	-2.285 ±0.723	0.397 ±0.177	-1.568 ±0.148	-5.688 ±0.805
NBD 557	MET 240	-0.920 ±0.197	0.711 ±0.489	-0.476 ±0.202	-0.416 ±0.110	-1.102 ±0.498
NBD 557	TRP 241	-2.046 ±0.383	0.008 ±0.218	0.117 ±0.098	-1.259 ±0.232	-3.181 ±0.514
NBD 557	GLY 286	-0.416 ±0.334	0.073 ±0.298	0.022 ±0.132	-0.274 ±0.285	-0.596 ±0.611
NBD 557	GLY 287	-0.941 ±0.282	-0.235 ±0.282	0.123 ±0.175	-0.910 ±0.241	-1.963 ±0.515
NBD 557	MET 289	-0.779 ±0.197	-0.012 ±0.215	-0.060 ±0.116	-0.584 ±0.111	-1.434 ±0.248

Table S4. Details of residuewise energy decompositions for interaction of antibody and gp120. All energy values are in kcal/mol. Data are shown as value ±standard error.

Total Energy Decomposition:

Resid 1	Resid 2	van der Waals	Electrostatic	Polar Solvation	Non-Polar Solv.	TOTAL
ASP 310	GLN 150	-0.208 ±0.194	-2.305 ±2.614	2.115 ±2.231	-0.182 ±0.227	-0.579 ±0.919
TYR 312	ARG 149	-1.448 ±0.332	-1.108 ±0.808	-0.166 ±0.578	-1.207 ±0.254	-3.928 ±0.832
TYR 312	LYS 235	-0.222 ±0.068	1.205 ±0.484	-1.765 ±0.404	-0.127 ±0.048	-0.909 ±0.282
TYR 312	GLN 236	-1.012 ±0.244	0.150 ±0.485	-0.446 ±0.316	-0.751 ±0.144	-2.059 ±0.450
LEU 329	ILE 237	-0.284 ±0.104	-0.033 ±0.013	0.066 ±0.016	-0.352 ±0.098	-0.604 ±0.164
ASP 331	ARG 233	1.160 ±0.969	-51.864 ±2.007	32.894 ±1.245	-0.536 ±0.035	-18.346 ±1.813
ASP 331	LYS 235	-0.204 ±0.050	-29.633 ±1.672	27.487 ±1.239	-0.087 ±0.033	-2.437 ±0.651
GLU 333	ARG 233	-0.984 ±0.314	-22.713 ±2.701	23.804 ±2.301	-1.013 ±0.135	-0.905 ±0.811
ASP 334	PRO 191	-0.731 ±0.194	-2.548 ±0.427	2.206 ±0.355	-0.605 ±0.134	-1.679 ±0.374
ASP 334	ARG 233	-0.011 ±0.621	-36.558 ±2.139	29.505 ±0.810	-0.553 ±0.062	-7.616 ±1.396
ASP 334	LYS 235	0.256 ±0.708	-43.911 ±2.874	34.074 ±1.058	-0.571 ±0.068	-10.152 ±1.845
ASP 336	ARG 233	-0.087 ±0.018	-22.654 ±0.875	21.651 ±0.640	-0.019 ±0.011	-1.109 ±0.337
ASP 336	LYS 235	0.507 ±0.620	-47.018 ±1.939	35.887 ±0.752	-0.376 ±0.037	-11.001 ±1.200
ASP 336	ILE 237	-1.215 ±0.244	2.300 ±0.830	-2.176 ±0.582	-0.937 ±0.143	-2.028 ±0.423
ASP 336	LYS 246	-0.050 ±0.631	-42.962 ±3.866	36.184 ±1.825	-0.690 ±0.136	-7.517 ±2.089
MET 338	VAL 31	-0.531 ±0.203	-0.033 ±0.143	0.042 ±0.110	-0.538 ±0.175	-1.060 ±0.359
MET 338	MET 248	-0.797 ±0.256	-0.192 ±0.220	0.065 ±0.093	-0.732 ±0.191	-1.656 ±0.441

Table S5. Clustering of gp120 with NBD without antibody

#Cluster	Frames	Frac	AvgDist	Stdev	Centroid	AvgCDist
0	10565	0.218	1.636	0.208	3429	2.281
1	5912	0.127	1.591	0.202	6565	2.560
2	4520	0.121	1.688	0.237	1663	2.357
3	3824	0.115	1.591	0.215	6002	2.515
4	2729	0.102	1.599	0.198	2063	2.378
5	566	0.079	1.516	0.219	1146	2.349
6	532	0.074	1.559	0.199	261	2.618
7	502	0.070	1.572	0.215	5254	2.301
8	479	0.053	1.524	0.204	4824	2.257
9	295	0.041	1.520	0.246	684	2.419

Table S6. Clustering of gp120 with NBD in N239G mutant.

#Cluster	Frames	Frac	AvgDist	Stdev	Centroid	AvgCDist
0	10803	0.180	1.578	0.164	3230	2.037
1	5569	0.157	1.528	0.174	7695	2.173
2	4512	0.151	1.551	0.177	6321	2.142
3	3368	0.137	1.533	0.172	9362	2.120
4	3040	0.104	1.497	0.151	5347	2.089
5	2731	0.073	1.535	0.189	2134	2.050
6	1692	0.069	1.600	0.210	780	2.225
7	511	0.051	1.511	0.194	1689	2.107
8	454	0.045	1.531	0.199	328	2.429
9	120	0.032	1.519	0.195	1365	2.158

Table S7. Clustering of gp120 with NBD in presence of antibody.

#Cluster	Frames	Frac	AvgDist	Stdev	Centroid	AvgCDist
0	10934	0.493	2.508	0.523	4357	10.009
1	5370	0.137	2.621	0.632	7213	5.879
2	4963	0.096	2.731	0.843	5298	7.652
3	3900	0.090	2.597	0.689	9238	5.369
4	2473	0.047	2.130	0.518	8553	6.321
5	1367	0.037	2.230	0.642	9822	6.619
6	310	0.031	2.300	0.668	9471	6.176
7	306	0.031	1.918	0.408	6263	5.332
8	283	0.028	1.909	0.478	6013	5.813
9	94	0.009	1.677	0.416	6830	6.540
